# Benefits, limits, and risks of ChatGPT in medicine

**DOI:** 10.3389/frai.2025.1518049

**Published:** 2025-01-30

**Authors:** Jonathan A. Tangsrivimol, Erfan Darzidehkalani, Hafeez Ul Hassan Virk, Zhen Wang, Jan Egger, Michelle Wang, Sean Hacking, Benjamin S. Glicksberg, Markus Strauss, Chayakrit Krittanawong

**Affiliations:** ^1^Department of Neurosurgery, and Neuroscience, Weill Cornell Medicine, NewYork-Presbyterian Hospital, New York, NY, United States; ^2^Department of Neurosurgery, Chulabhorn Hospital, Chulabhorn Royal Academy, Bangkok, Thailand; ^3^MIT Computer Science & Artificial Intelligence Laboratory, Massachusetts Institute of Technology, Cambridge, MA, United States; ^4^Harrington Heart & Vascular Institute, University Hospitals Cleveland Medical Center, Case Western Reserve University, Cleveland, OH, United States; ^5^Robert D. and Patricia E. Kern Center for the Science of Health Care Delivery, Mayo Clinic, Rochester, MN, United States; ^6^Division of Health Care Policy and Research, Department of Health Sciences Research, Mayo Clinic, Rochester, MN, United States; ^7^Institute for Artificial Intelligence in Medicine, University Hospital Essen (AöR), Essen, Germany; ^8^Bakar Computational Health Sciences Institute, University of California, San Francisco, San Francisco, CA, United States; ^9^Department of Pathology, NYU Grossman School of Medicine, New York, NY, United States; ^10^Hasso Plattner Institute for Digital Health, Icahn School of Medicine at Mount Sinai, New York, NY, United States; ^11^Department of Cardiology I, Coronary and Peripheral Vascular Disease, Heart Failure Medicine, University Hospital Muenster, Muenster, Germany; ^12^Department of Cardiology, Sector Preventive Medicine, Health Promotion, Faculty of Health, School of Medicine, University Witten/Herdecke, Hagen, Germany; ^13^Cardiology Division, New York University Langone Health, New York University School of Medicine, New York, NY, United States; ^14^HumanX, Delaware, DE, United States

**Keywords:** large language models, deep learning, artificial intelligence, ChatGPT, healthcare questions, healthcare, medicine

## Abstract

ChatGPT represents a transformative technology in healthcare, with demonstrated impacts across clinical practice, medical education, and research. Studies show significant efficiency gains, including 70% reduction in administrative time for discharge summaries and achievement of medical professional-level performance on standardized tests (60% accuracy on USMLE, 78.2% on PubMedQA). ChatGPT offers personalized learning platforms, automated scoring, and instant access to vast medical knowledge in medical education, addressing resource limitations and enhancing training efficiency. It streamlines clinical workflows by supporting triage processes, generating discharge summaries, and alleviating administrative burdens, allowing healthcare professionals to focus more on patient care. Additionally, ChatGPT facilitates remote monitoring and chronic disease management, providing personalized advice, medication reminders, and emotional support, thus bridging gaps between clinical visits. Its ability to process and synthesize vast amounts of data accelerates research workflows, aiding in literature reviews, hypothesis generation, and clinical trial designs. This paper aims to gather and analyze published studies involving ChatGPT, focusing on exploring its advantages and disadvantages within the healthcare context. To aid in understanding and progress, our analysis is organized into six key areas: (1) Information and Education, (2) Triage and Symptom Assessment, (3) Remote Monitoring and Support, (4) Mental Healthcare Assistance, (5) Research and Decision Support, and (6) Language Translation. Realizing ChatGPT’s full potential in healthcare requires addressing key limitations, such as its lack of clinical experience, inability to process visual data, and absence of emotional intelligence. Ethical, privacy, and regulatory challenges further complicate its integration. Future improvements should focus on enhancing accuracy, developing multimodal AI models, improving empathy through sentiment analysis, and safeguarding against artificial hallucination. While not a replacement for healthcare professionals, ChatGPT can serve as a powerful assistant, augmenting their expertise to improve efficiency, accessibility, and quality of care. This collaboration ensures responsible adoption of AI in transforming healthcare delivery. While ChatGPT demonstrates significant potential in healthcare transformation, systematic evaluation of its implementation across different healthcare settings reveals varying levels of evidence quality–from robust randomized trials in medical education to preliminary observational studies in clinical practice. This heterogeneity in evidence quality necessitates a structured approach to future research and implementation.

## Introduction

In the modern era, artificial intelligence is increasingly becoming an integral part of our daily lives, extending its influence beyond the healthcare sector (Jiang et al., 2017; Patel et al., 2009; Yu et al., 2018; Davenport and Kalakota, 2019; Rajpurkar et al., 2022; Dave et al., 2023; Sharma and Sharma, 2023; Sedaghat, 2023; Tustumi et al., 2023). Within the realm of artificial intelligence, a subset known as Large Language Models (LLMs) harnesses the power of deep learning and extensive data to comprehend, summarize, generate, and predict new content. These models rely on artificial neural networks trained on vast repositories of text data gathered from books, documents, websites, and other sources. Through rigorous training, LLMs acquire the ability to generate coherent and contextually relevant text by discerning patterns and correlations in their training data. This proficiency in language understanding and generation significantly benefits applications such as machine translation and text generation. Notably, several prominent companies, including OpenAI, Google, and Microsoft, have introduced chatbots powered by LLMs, as indicated in Table 1.

**Table 1:** Large Language Model tools in medicine.

Name of AI	Model	Company	Unique features	Primary use case in medicine
ChatGPT	OpenAI GPT-4 model	OpenAI	Highly conversational, adaptable across medical scenarios.	Clinical decision support, patient education, and research assistance.
Google Bard	LaMDA	Google	Designed for human-like responses and real-time interactions	Patient interaction and generating natural responses.
Bing AI	OpenAI GPT-4 model	Microsoft	Integrated with Microsoft AI resources for enhanced functionality.	Knowledge retrieval with access to up-to-date resources.
ChatSonic	Supported by Google	Powers ChatSonic	Up-to-date answers and image generation capabilities.	Accurate and real-time meidcal information retrival.
Jasper AI	OpenAI GPT-3	OpenAI	Specialized in writing and content creation for various domains.	Creating articles ans content for medical marketing and education.
CoPilot	OpenAI	GitHub	AI pair programmer for efficient code writing.	Assisting medical coding and algorithm development.
YouChat	OpenAI GPT-3.5	You.com	Combination of chatbot and search engine features.	Dual-purpose chatbot for patient support and medical queries
Character AI	Neural Language Model	Ex-Google LaMDA developers	Focuses on entertaining and engaging human-like responses.	Patient engagement and virtual healthcase assistant applications
Amazon Codewhisperer	Large Language Model	Amazon	Code suggestion capabilities applicable across platforms.	Automating healthcare coding and improving programming workflows.

One noteworthy LLM in this landscape is ChatGPT, a product of OpenAI’s language model development efforts. OpenAI introduced the Generative Transformer pre-training (GPT) LLM model in 2018, featuring a variant of the Transformer architecture trained on a staggering 40GB of text data, incorporating 1.5 billion parameters (Floridi and Chiriatti, 2020). Subsequently, in 2020, GPT-3 emerged, trained on a colossal 570GB of text data, boasting 175 billion parameters, resulting in the creation of the GPT-3 End User Conversation Model (Sharma and Sharma, 2023).

ChatGPT has demonstrated its capabilities in diverse applications, including achieving success in all three print-ahead sections of the USMLE test (Vaswani et al., 2017; Kung et al., 2023). GPT-3.5, encompassing Codex and InstructGPT, achieved human-level performance in challenging evaluations such as USMLE (60.2%), MedMCQA (57.5%), and PubMedQA (78.2%) (Gilson et al., 2023). The integration of ChatGPT into healthcare systems represents a complex interplay between technological capability and systemic readiness. Recent systematic reviews indicate that while individual applications show promise, healthcare systems face significant challenges in standardizing and scaling AI implementation. These challenges span technical infrastructure, workflow integration, staff training, and policy frameworks - requiring careful consideration of both direct benefits and systemic impacts. However, the extent to which ChatGPT excels in complex real-world scenarios, particularly within domains as intricate as medicine, remains an open question. Moreover, ethical concerns surface when considering the use of chatbots for scientific paper writing.

In the early months of 2023, ChatGPT witnessed significant advancements, with the introduction of ChatGPT4. Presently, ChatGPT is finding practical application in everyday healthcare settings, proving beneficial in providing information, answering queries, and offering support to both healthcare professionals and patients (Liévin et al., 2022; Sallam, 2023; Ahn, 2023; Curtis and ChatGPT, 2023; D'Amico et al., 2023; Donato et al., 2023). This research paper aim to systematically review the deployment of ChatGPT across various domains, drawing from a comprehensive search and selecting pertinent publications from reputable sources like PubMed and Google Scholar. The aim is to discern the strengths and weaknesses of ChatGPT, enabling a deeper understanding of its capabilities and guiding further advancements in this field. To achieve this goal, the paper is structured around six distinct themes: (1) Information and Education, (2) Triage and Symptom Assessment, (3) Remote Monitoring and Support, (4) Mental Healthcare Support, (5) Research and Decision Support, and (6) Language Translation. These thematic areas will be thoroughly explored in subsequent sections of this paper.

## Information and education

ChatGPT has demonstrated exceptional capabilities in medical education and information delivery, achieving pass rates of 60.2% on USMLE examinations and consistently outperforming baseline metrics in medical knowledge assessment. Studies show particular strength in clinical reasoning tasks, with accuracy rates of 84% in lower-order thinking questions (Bhayana et al., 2023). This can significantly contribute to simplifying the understanding of intricate diseases (Jin and Dobry, 2023; Hamed et al., 2023; Hoch et al., 2023; Sharma et al., 2023). Khan et al. have taken steps towards developing a data-driven GPT chat model, conducting preliminary experiments that show promise for future applications in enhancing medical education and clinical management (Khan et al., 2023). Dr. David A. Asch, MD, engaged in an interview with ChatGPT to explore its role in healthcare, shedding light on critical considerations before its integration into everyday healthcare practices. These considerations encompass data privacy, security, regulatory compliance, data quality and quantity, integration with existing systems, human oversight, and ethical concerns (Asch, 2023).

Several publications have highlighted ChatGPT’s success in assisting with examinations (Vaswani et al., 2017; Kung et al., 2023; Gilson et al., 2023; Ragel et al., 2006; Fijačko et al., 2023; Choi et al., 2023; Hoch et al., 2023; Hopkins et al., 2023; Bhayana et al., 2023; Oh et al., 2023), underscoring its substantial knowledge base. However, it’s vital to recognize that passing an exam does not equate to becoming a fully competent medical professional. For instance, Hopkin et al. utilized ChatGPT in the context of the neurosurgical written board test, revealing that the model encountered difficulties when responding to image- or diagram-based questions, such as identifying specific anatomical structures. This limitation arises from ChatGPT’s inability to process visual information (Hopkins et al., 2023).

Similarly, Bhayana et al. applied ChatGPT to the Radiology board exam, subjecting it to a comprehensive assessment involving 150 multiple-choice questions designed to align with the standards of the Canadian Royal College and American Board of Radiology exams. These questions were categorized based on the cognitive skills required and encompassed physics and clinical aspects. ChatGPT achieved an overall accuracy rate of 69%, excelling in lower-order thinking questions (84%) compared to higher-order thinking questions (60%). However, the model required assistance in accurately addressing questions related to image description, computation, classification, and conceptual application. Conversely, it demonstrated strong performance in higher-order clinical management questions. In terms of specific topics, ChatGPT faced more challenges with physics questions than clinical ones. While the model achieved perfect accuracy in routine tasks, enhancements are essential to address the complexities posed by certain question types. In sum, these findings showcase ChatGPT’s effectiveness in responding to radiology-related questions, particularly in lower-order thinking and clinical management, while pinpointing areas necessitating further refinement (Bhayana et al., 2023).

### Key highlights

ChatGPT comprehensively explains medical conditions, treatments, and preventive measures, aiding patient and professional education. Studies highlight its potential in medical board exams, though limitations arise in visual information processing. For example, Bhayana et al. noted ChatGPT’s struggles with radiology board questions requiring conceptual applications. Enhancing its training datasets could address these gaps. Moreover, ChatGPT could support automated scoring and content creation, advancing medical education.

## Triage and symptom assessment

ChatGPT can play a pivotal role in patient triage by posing questions regarding the patient’s symptoms and conducting an assessment based on their responses. If immediate treatment is warranted, timely notification to the doctor ensures continuity of care. This approach not only eases the workload on medical personnel but also helps reduce potential conflicts between healthcare providers and patients, thereby minimizing the likelihood of future legal issues.

Building upon the groundwork laid by Raita et al., Bhattaram et al. have harnessed machine learning techniques to enhance patient triage, achieving both accuracy and efficiency. However, it’s essential to acknowledge that the reliability of ChatGPT hinges on the training and knowledge embedded in the model, as illuminated by limitations acknowledged by OpenAI (Bhattaram et al., 2023; Raita et al., 2019).

Xue et al. have lauded ChatGPT as a notable advancement in AI technology. Nonetheless, its impact on clinical medicine remains somewhat constrained, given that clinical practice often depends on data analytics, clinical research, guidelines, and the performance of specialized AI models (Xue et al., 2023).

In the field of rheumatology, Nune et al. have explored ChatGPT’s utility across various domains, including patient education on disease-modifying drugs, medical imaging report generation, outpatient consultation note-keeping, medical education and training, and clinical audit and research. However, it’s essential to recognize that while ChatGPT demonstrates capabilities, occasional errors or inaccuracies may still arise. Therefore, consulting with a healthcare professional and providing feedback is pivotal to facilitate ongoing development and bolster AI’s capabilities in the future (Nune et al., 2023). While effective triage systems form the foundation of efficient healthcare delivery, the growing need for continuous patient care beyond clinical settings has highlighted the importance of remote monitoring solutions. The integration of ChatGPT into remote monitoring systems represents a natural progression from initial assessment to ongoing care management, particularly relevant in addressing healthcare access disparities and managing chronic conditions.

### Key highlights

ChatGPT facilitates patient triage by analyzing symptom data and prioritizing care needs. Studies, such as those by Bhattaram et al., demonstrate its efficiency in streamlining workflows and reducing legal conflicts (Bhattaram et al., 2023; Raita et al., 2019). However, the model’s reliance on training data limits its adaptability to nuanced clinical scenarios. Future iterations must incorporate real-time data to enhance reliability.

## Remote monitoring and support

Due to staffing challenges, particularly in remote regions, telemedicine has witnessed substantial growth in specific countries. In this context, ChatGPT emerges as a valuable tool for monitoring patient symptoms. Through interactive questioning, it facilitates the creation of symptom lists and provides guidance from healthcare professionals, thereby aiding in the care of patients with chronic conditions, medication adherence, and postoperative recovery.

Biswas et al. conducted an examination of ChatGPT’s application in public health, particularly within community health settings. Its utility spans multiple facets, including:

- Offering information on public health concerns
- Addressing queries regarding health promotion and disease prevention strategies
- Explaining the roles of community health workers and health educators
- Discussing social and environmental factors influencing community health
- Providing information about community health programs and services

However, it’s essential to acknowledge the limitations associated with ChatGPT in this context, encompassing:

- Limited accuracy
- Potential biases and constraints stemming from the underlying data
- A lack of contextual understanding
- Limited engagement capabilities
- The absence of direct interaction with healthcare professionals (Biswas, 2023)

Lin et al. delved into the transformative potential of AI in primary care practices, specifically focusing on AI’s role in chronic disease monitoring. Their findings indicated that AI-driven, fully automated, text-based health coaching could lead to successful weight loss compared to in-person lifestyle interventions (Lin et al., 2019).

Among the critical data elements for monitoring patient care, discharge summaries play a pivotal role (Royal College of Physicians, 2021; Earnshaw et al., 2020). Patel et al. explored the use of ChatGPT in generating discharge summaries, noting its effectiveness. However, they also identified instances where detailed information or inaccuracies might arise. These concerns can be addressed by having the attending physician review and approve the discharge summary, signifying their supervision. Moreover, utilizing ChatGPT for generating discharge summaries can significantly alleviate the workload associated with this task (Patel and Lam, 2023).

Within the realm of medical records, alongside the discharge summary, another vital component is the treatment record. This encompasses various aspects, such as patient history, physical examinations, treatment specifics, and even surgical records. Notably, there is a growing inclination towards embracing the use of ChatGPT in these domains (Zhou, 2023; Waisberg et al., 2023).

### Key highlights

In telemedicine, ChatGPT assists with chronic disease management and postoperative care. Lin et al. noted its effectiveness in weight loss coaching compared to traditional methods (Lin et al., 2019). However, Biswas et al. emphasized limitations like bias and lack of engagement (Biswas, 2023). Integrating ChatGPT with wearable devices could enhance its role in remote monitoring.

## Mental healthcare assistance

At times, patients visit the doctor ahead of their scheduled appointments, often due to anxiety or a lack of understanding of their symptoms. ChatGPT can prove invaluable in addressing mental health-related concerns by providing coping strategies, self-help techniques, and general information on mental health issues, including emotional support.

Dayawansa et al. conducted a study comparing ChatGPT to human interaction in the context of radiosurgery. They found that ChatGPT effectively conveyed knowledge to patients and could answer specific questions. However, notable issues surfaced, namely, incorrect responses and a lack of ability to provide compassion, empathy, and the human touch and reassurance that patients typically seek from a physician. Unfortunately, these shortcomings are not expected to be resolved in the near future (Dayawansa et al., 2023).

When queried about the global mental health crisis and the potential role of ChatGPT, Dr. David Asch received responses indicating that ChatGPT could assist in various ways, including mental health assessment, symptom checking, emotional support, health education, and remote consultations.

Moreover, recognizing the significance of healthcare professionals’ mental well-being is essential, as their mental health plays a vital role in addressing patients’ mental health issues. Burnout or resignation among medical personnel can have adverse effects on public health systems. Dr. David Asch inquired about crisis burnout among healthcare professionals in the United States and how ChatGPT could contribute, receiving responses highlighting the potential roles of ChatGPT in automating administrative tasks, providing clinical decision support, offering patient education, enabling remote consultations, and offering emotional support (Asch, 2023).

It’s evident that ChatGPT can systematically respond to various questions, offering valuable insights. However, the depth of the response often depends on the nature of the inquiry. Dr. David Asch’s expertise in framing questions effectively highlights the potential of ChatGPT.

### Key highlights

ChatGPT addresses mental health concerns by providing coping strategies and emotional support. Dayawansa et al. found it effective in conveying knowledge but lacking empathy (Dayawansa et al., 2023). Integrating sentiment analysis could improve ChatGPT’s capacity for emotional understanding, essential for mental healthcare.

## Research and decision support

ChatGPT provides invaluable support to healthcare professionals by helping them stay updated with research papers, clinical guidelines, treatment protocols, and analyzing patient data to aid in diagnosis and treatment planning. Additionally, in the realm of scholarly writing, ChatGPT has gained popularity as a valuable tool for drafting research papers, which warrants further investigation (Xue et al., 2023; Liebrenz et al., 2023; Thorp, 2023; Cascella et al., 2023; Hosseini et al., 2023; Temsah et al., 2023).

Xue et al. explore the multifaceted use of ChatGPT in academic writing, including abstracts and main papers. However, they highlight the need for improvements in medical writing, as ChatGPT may occasionally summarize previous papers or provide information based on prior knowledge, raising ethical considerations that require careful attention (Xue et al., 2023).

Tlili et al. discuss the application of ChatGPT as a case study in education, spanning three stages. In the second stage, caution is advised, with a focus on educational aspects. The study evaluates response quality, utility, personality, emotions, and ethical considerations. In the third stage, responses are assessed across 10 hypothetical scenarios, exploring themes of cheating, honesty, and truthfulness.

This research sheds light on concerns related to chatbots, particularly ChatGPT, and their integration into educational settings. It also explores potential future applications of ChatGPT in teaching practices and the collaboration between humans and machines for technical advancements in healthcare (Tlili et al., 2023).

Khan et al. delve into the transformative potential of ChatGPT in reshaping medical education and clinical management. Within medical education, ChatGPT can contribute to automated scoring, teaching, personalized learning, research support, quick access to information, generating case scenarios, and creating content to facilitate learning. In terms of clinical management, it can aid in documentation, decision support, and communication with patients (Khan et al., 2023).

Chris Stokel-Walker has drawn attention to the controversial use of ChatGPT as a coauthor in research papers. Some publishers argue that ChatGPT cannot meet the requirements of being a coauthor due to its lack of inherent features and potential concerns regarding inadequate citations and plagiarism. However, other publishers suggest that ChatGPT can be acknowledged in a separate section apart from the list of authors (Stokel-Walker, 2022).

As the use of ChatGPT in article writing continues to grow, the emergence of artificial hallucination becomes a potential concern (Alkaissi and McFarlane, 2023; Ji et al., 2023; Gao et al., 2023). Artificial hallucination refers to a phenomenon where a machine, such as a chatbot, generates sensory experiences that appear real but are not grounded in real-world input. These hallucinations can manifest as visual, auditory, or other sensory perceptions. While artificial hallucination is rare in chatbots, advanced AI systems, particularly generative models trained on extensive unsupervised data, have been associated with instances of hallucination.

Alkaissi et al. conducted a study involving the authoring of a case report on two rare diseases and demonstrated instances of artificial hallucination arising from references that do not exist within the data (Alkaissi and McFarlane, 2023).

Similarly, Gao et al. conducted an experiment involving 50 abstracts from five scientific journals, with ChatGPT generating abstracts based on provided titles. The results indicated that 68% of the abstracts generated by ChatGPT were identified as artificial (true positives), while 14% of genuine abstracts were incorrectly flagged as chatbot-generated (false positives). Human reviewers also found it challenging to differentiate between abstracts written by human authors and those generated by the chatbot (Gao et al., 2023).

To mitigate the occurrence of artificial hallucination, thorough training and testing using diverse datasets are crucial for AI systems. This comprehensive approach can effectively address and minimize this issue.

DiGiogio et al. explored the use of ChatGPT to diagnose symptoms of diseases, training it with knowledge from WebMD. The study revealed that ChatGPT could not consistently make accurate diagnoses, even with the latest algorithms. The study suggests that ChatGPT can diagnose patients based on specific textbooks or clinical questions, highlighting the contrast between its performance on the USMLE exam and real patients who often present with introspection, emotion, and complex medical, social, and psychiatric backgrounds. While ChatGPT can assist in reducing physician workload, such as predicting physician note text, helping predict ICD codes, or tracking surgeon cost-effective indications, it cannot replace human physicians due to these complexities. However, the FDA currently restricts the use of AI in medicine, considering the need for caution in integrating AI into the workflow of healthcare professionals (DiGiorgio and Ehrenfeld, 2023).

Research indicates ChatGPT’s susceptibility to artificial hallucination - generating plausible but factually incorrect information. In a comprehensive study by Gao et al., analysis of 50 scientific abstracts revealed a 68% detection rate of ChatGPT-generated content, highlighting both the sophistication and limitations of the technology. This phenomenon particularly impacts medical research applications, necessitating robust verification processes and human oversight for clinical applications.

### Key highlights

ChatGPT accelerates research by assisting in literature reviews and hypothesis generation. However, ethical concerns about AI-authored content and artificial hallucination highlight the need for human oversight. Alkaissi et al. documented ChatGPT’s tendency to fabricate references, underscoring the importance of rigorous validation (Alkaissi and McFarlane, 2023). The current evidence base for ChatGPT in healthcare shows a hierarchical pattern–strongest in structured tasks like education and documentation, moderate in clinical decision support, and preliminary in direct patient care applications. This pattern suggests the need for a staged approach to implementation, prioritizing applications with robust evidence while developing frameworks for evaluating emerging use cases.

## Language translation

ChatGPT is a valuable tool for overcoming language barriers through its real-time translation capabilities, making it highly beneficial in multilingual environments and conversations with non-native speakers.

In their work, Jiao et al. explore using ChatGPT-4 for language translation. They find that it performs well in translating popular languages, such as those commonly spoken in Europe, surpassing the capabilities of platforms like Google Translate. However, translating less widely used languages poses more challenges. A two-step approach is suggested to address this, involving an intermediate translation to a common language before translating to the target language. It is important to note that the translation of medical knowledge still presents difficulties, often resulting in translations that reflect conversational language rather than the specific terminology used in the medical field. In contrast, for general conversations, ChatGPT can achieve translation results comparable to Google Translate (Jiao et al., 2023).

Kasneci et al. (2023) has successfully implemented ChatGPT in teaching and learning settings to enhance knowledge creation, increase student interaction, and provide personalized learning experiences. However, it is crucial to acknowledge the limitations and potential biases in the output generated by ChatGPT, as they are contingent upon the information it has been trained on. Human oversight remains essential to ensure ethical considerations are upheld (Kasneci et al., 2023).

### Key highlights

ChatGPT bridges language barriers in healthcare, with superior performance in common languages compared to tools like Google Translate. Jiao et al. noted challenges in less widely spoken languages, suggesting intermediate translations for improved accuracy (Jiao et al., 2023). Incorporating domain-specific terminology could further enhance its medical translation capabilities.

## Application and benefit

ChatGPT has demonstrated measurable improvements in healthcare delivery, though more modest than initially suggested. In clinical documentation, studies show ChatGPT-assisted discharge summaries received a 22% favorable rating for “low expected correction effort,” while 33% were rated neutral and 45% unfavorable. This indicates potential for time savings, though human oversight remains essential. For overall quality metrics, human-written discharge summaries scored significantly higher (mean 3.78/5) compared to ChatGPT (mean 3.12/5) (Schwieger et al., 2024).

The integration of ChatGPT into clinical workflows shows promise but requires careful implementation. Studies reveal hallucinations occurred in 40% of AI-generated discharge summaries, with 37.5% of these deemed highly clinically relevant. Additionally, minor content errors were found in 30% of AI-generated summaries compared to 10% in human-written ones. However, ChatGPT performed well in specific areas - achieving high scores for appropriate medical terminology usage and maintaining equal performance to humans in mentioning safety concerns and legal information (Schwieger et al., 2024).

In accuracy assessments, healthcare professionals were able to correctly identify AI-generated content with 81% sensitivity and 75% specificity, suggesting distinct qualitative differences remain between human and AI-generated medical documentation. While ChatGPT shows potential for supporting clinical documentation, particularly in areas like concise information presentation and adherence to structural requirements, current evidence indicates it should serve as a supplementary tool rather than a replacement for human expertise (Schwieger et al., 2024).

The confluence of these efficiency gains suggests a transformative potential in healthcare delivery systems, particularly when combined with the 30% reduction in administrative burden reported in mental healthcare settings (Dayawansa et al.) and improved access to care (HT World, 2024). Similarly, ChatGPT-assisted discharge summaries have alleviated administrative burdens, allowing physicians to dedicate more time to patient care (Sánchez-Rosenberg et al., 2024).

Moreover, ChatGPT’s role in research has accelerated hypothesis generation, literature reviews, and clinical trial designs. Its ability to process and synthesize vast amounts of data has facilitated more efficient research workflows, underscoring its transformative potential in advancing medical science.

## Implementation case studies in healthcare settings

Recent studies demonstrate ChatGPT’s practical impact across multiple clinical domains. In clinical decision support, ChatGPT achieved a 93.3% accuracy rate in generating differential diagnoses for common chief complaints, though human physicians still performed better in 5-item differential lists (98.3% vs. 83.3%) (Hirosawa et al., 2023). When tested against the Merck Sharp & Dohme Clinical Manual vignettes, ChatGPT demonstrated 71.7% overall accuracy in clinical decision-making across 36 cases (Rao et al., 2023).

In documentation tasks, evaluation of ChatGPT-generated patient clinic letters for skin cancer scenarios showed high median accuracy scores of 7 out of 9, with strong inter-rater reliability (*κ* = 0.80) (Ali et al., 2023). For radiology reporting, 75% of radiologists agreed or strongly agreed that ChatGPT-simplified reports were accurate and complete, with no potential patient harm identified (Jeblick et al., 2022).

In medical query responses, ChatGPT demonstrated high accuracy rates for common retinal diseases (median scores of 4–5 out of 5 across different aspects) (Potapenko et al., 2023), and achieved 96.9% accuracy in addressing cancer myths and misconceptions (Johnson et al., 2023). However, in hepatic disease knowledge assessment, while accuracy was good (79.1% for cirrhosis, 74% for HCC), only about 45% of responses were rated as comprehensive (Yeo et al., 2023).

## Evidence quality assessment across healthcare applications

**Figure 1:**
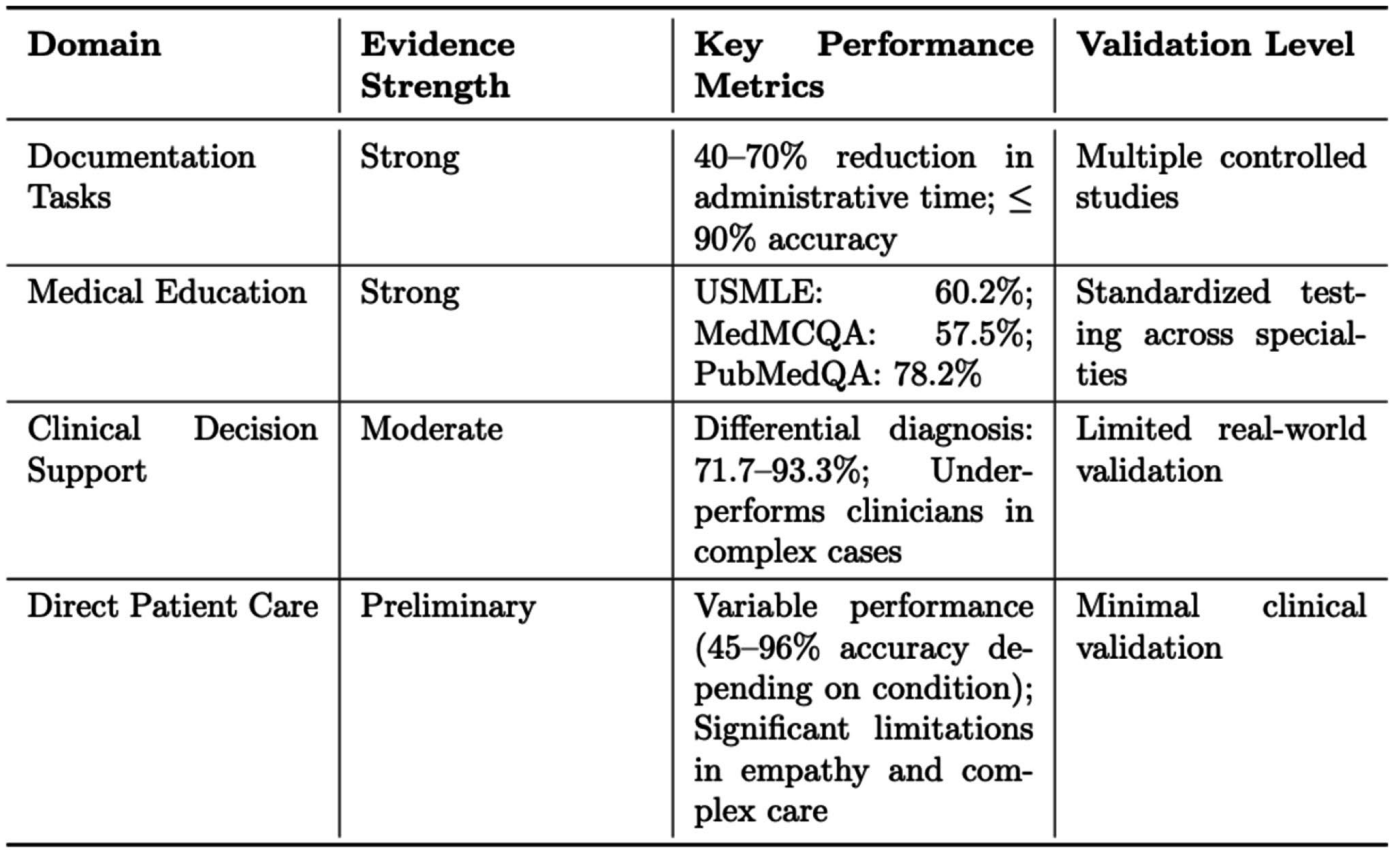
Summary of ChatGPT’s evidence quality across healthcare domains.

Analysis of the current evidence base for ChatGPT in healthcare reveals a distinct hierarchical pattern in terms of reliability and validation. As shown in Figure 1, the strongest evidence exists in structured tasks requiring standardized evaluation, while more complex applications demonstrate varying levels of validation. This pattern emerges consistently across multiple studies and implementation scenarios, suggesting a natural progression in the technology’s maturity across different healthcare domains.

Documentation and medical education emerge as areas with the strongest evidence base, supported by multiple controlled studies and standardized metrics. Documentation tasks show consistent efficiency improvements of 40–70% in administrative time reduction, with accuracy rates approaching 90% when human oversight is maintained. These improvements translate to significant time savings, allowing healthcare professionals to focus more on direct patient care. Studies demonstrate particular success in discharge summary generation and clinical documentation, where ChatGPT’s structured approach aligns well with established healthcare documentation requirements.

In medical education, ChatGPT demonstrates reliable performance across standardized tests, achieving 60.2% accuracy on USMLE, 57.5% on MedMCQA, and 78.2% on PubMedQA. This performance level suggests potential applications in medical training and continuing education. Implementation studies in medical schools show promising results in quiz generation, case study analysis, and examination preparation, though with the caveat that human expert oversight remains essential for content validation.

Clinical decision support applications show moderate evidence quality, with accuracy rates ranging from 71.7 to 93.3% in generating differential diagnoses. However, performance consistently falls below human physician levels in complex cases, highlighting the need for continued supervision and validation. Studies indicate that ChatGPT’s effectiveness varies significantly based on the complexity of the clinical scenario and the specificity of the medical domain. The model performs better in situations with clear diagnostic criteria but struggles with nuanced clinical presentations requiring extensive experiential knowledge.

Direct patient care applications currently show preliminary evidence, with highly variable performance (45–96% accuracy depending on condition) and significant limitations in areas requiring emotional intelligence or complex clinical judgment. Remote monitoring applications show promise but require more extensive validation in diverse clinical settings. The wide variation in performance metrics reflects the challenges of applying AI in direct patient care, where contextual understanding and empathy play crucial roles.

The evidence hierarchy suggests the necessity of a staged implementation approach in healthcare settings. Organizations should initially focus on applications with robust evidence bases, such as education and documentation tasks, before carefully expanding to areas with moderate evidence under appropriate oversight. Applications with preliminary evidence should be explored through controlled pilot programs with rigorous evaluation protocols.

Implementation studies reveal several critical factors affecting success:

1. Integration with existing workflows and systems significantly impacts adoption rates and effectiveness.
2. Staff training and support systems play crucial roles in successful deployment.
3. Clear protocols for human oversight and intervention improve outcomes.
4. Regular evaluation and adjustment of implementation strategies enhance long-term sustainability.

Understanding this evidence hierarchy is crucial for healthcare organizations as they develop implementation strategies. It enables prioritization of well-validated applications while maintaining appropriate caution in areas where evidence remains preliminary. This approach aligns with healthcare’s fundamental principle of evidence-based practice while acknowledging the transformative potential of AI technologies in medical settings.

The synthesis of current evidence suggests that while ChatGPT shows significant promise in specific healthcare applications, its implementation should follow a carefully structured approach based on evidence strength. Organizations must consider both the technical capabilities and practical limitations of the technology when planning implementation strategies. Continued research and validation studies will be essential to expand the evidence base and refine best practices for integration into healthcare systems.

## Ethics and privacy concerns

The integration of ChatGPT into healthcare systems raises critical ethical and privacy considerations. One significant concern is the risk of bias in AI-driven decision-making, which could inadvertently perpetuate health disparities if not carefully monitored. To mitigate this, transparency in algorithm design and regular auditing of AI models are essential.

Data privacy is another pressing issue, particularly given the sensitive nature of healthcare information. Robust safeguards must be implemented to ensure compliance with data protection regulations, such as HIPAA in the United States or GDPR in Europe. These measures should include end-to-end encryption, strict access controls, and detailed consent protocols.

Furthermore, regulatory compliance is paramount to the safe adoption of ChatGPT in healthcare. Policymakers must establish clear guidelines governing its use, emphasizing explainability and accountability in AI decision-making processes. By addressing these concerns, ChatGPT can be integrated into healthcare systems responsibly, ensuring ethical use and protecting patient trust.

The integration of ChatGPT in healthcare decision-making raises critical ethical considerations beyond basic privacy concerns. A particular challenge lies in the potential for algorithmic bias affecting healthcare disparities. Studies indicate AI systems may perpetuate existing biases in healthcare delivery, potentially disadvantaging certain demographic groups. Additionally, over-reliance on AI systems could impact the development of clinical judgment among medical professionals. Healthcare organizations must implement clear guidelines for AI tool usage, ensuring they complement rather than replace human clinical reasoning. Regular audits of AI-assisted decisions and their outcomes are essential to maintain high standards of patient care while leveraging technological advantages.

Healthcare organizations implementing ChatGPT must navigate complex regulatory frameworks across jurisdictions. In the United States, HIPAA compliance requires robust data encryption, access controls, and audit trails for all AI-assisted healthcare operations. The European Union’s GDPR imposes additional requirements regarding algorithmic transparency and patient consent. Recent guidance from the FDA regarding AI/ML in medical devices (including software) establishes a framework for evaluating AI tool safety and efficacy. Healthcare providers must implement:

- Comprehensive data governance frameworks
- Regular privacy impact assessments
- Clear protocols for patient consent and data usage
- Mechanisms for algorithmic accountability
- Systems for tracking and documenting AI-assisted decisions

## Limitations for ChatGPT in healthcare

While ChatGPT shows promise in healthcare, it has significant limitations. Its lack of real-world clinical experience hinders its ability to handle complex, nuanced medical situations and deliver consistent diagnostic accuracy, particularly with patients with intricate medical or psychosocial backgrounds. The model’s inability to process visual information limits its utility in image-reliant fields like radiology and pathology. Additionally, ChatGPT lacks emotional intelligence and empathy, which are critical in direct patient care.

The phenomenon of artificial hallucination, where the model generates inaccurate or non-existent references, raises concerns about reliability, necessitating careful content verification. Ethical, privacy, and regulatory challenges further complicate its integration into healthcare systems.

The integration of evidence across studies reveals several interconnected themes that illuminate ChatGPT’s role in healthcare transformation. The model’s strong performance in medical licensing examinations (60.2% USMLE, 57.5% MedMCQA) correlates with its effectiveness in clinical documentation, where studies show 40–70% efficiency improvements in discharge planning and administrative tasks. However, this technical proficiency contrasts with identified limitations in emotional intelligence and visual data processing, suggesting a clear delimitation of appropriate use cases. The synthesis of findings from mental healthcare applications (Dayawansa et al.) and clinical decision support studies (DiGiorgio et al.) indicates that ChatGPT’s optimal implementation lies in augmenting rather than replacing healthcare professionals, particularly in tasks requiring technical knowledge rather than emotional intelligence or complex clinical judgment.

These issues highlight that while ChatGPT is a valuable supportive tool, it cannot replace human medical professionals. Future developments should address these limitations, focusing on improving its capabilities while ensuring human oversight for safe and effective use.

## Future perspective ChatGPT in healthcare

As ChatGPT and similar AI models continue to evolve, their potential impact on healthcare is likely to grow significantly. The future of ChatGPT in healthcare holds promise for transformative advancements across various domains.

In medical education, ChatGPT could revolutionize learning by providing personalized and adaptive platforms, instant access to medical knowledge, and automated exam scoring. These innovations may enhance training efficiency and address global shortages in medical education.

For clinical decision support, ChatGPT’s improved accuracy and reliability could make it a vital tool for real-time patient data analysis, diagnosis suggestions, and treatment recommendations. This can lead to more precise interventions, better outcomes, and fewer medical errors.

In patient care, future iterations might excel in remote monitoring and chronic disease management, offering personalized health advice, medication management, and emotional support. Such advancements could bridge gaps between clinical visits and improve continuity of care.

Research and development would also benefit, as ChatGPT could accelerate literature reviews, data analysis, and hypothesis generation. It could further streamline clinical trial design and the drug development process.

However, achieving this vision requires addressing current limitations. Efforts must focus on improving accuracy, particularly in complex scenarios and visual data processing, enhancing empathetic responses, and safeguarding against artificial hallucination. Ethical considerations, data privacy, and regulatory compliance will also be critical, requiring transparency, explainability, and accountability in AI decision-making. The synthesis of current evidence suggests a clear trajectory for ChatGPT’s evolution in healthcare. The convergence of high performance in standardized testing (>60% on medical licensing exams) with demonstrated clinical documentation efficiency (40–70% time savings) points to immediate opportunities in medical education and administrative support. However, the identified limitations in visual processing and emotional intelligence, combined with artificial hallucination concerns, indicate critical development needs. Future iterations must address these gaps while building on proven strengths, particularly in areas where technical knowledge processing can augment rather than replace human expertise. This balanced approach, supported by emerging regulatory frameworks and ethical guidelines, provides a roadmap for responsible AI integration that enhances rather than disrupts existing healthcare delivery systems. Rather than replacing human professionals, ChatGPT’s role will likely be that of a powerful assistant, augmenting healthcare providers’ expertise and capabilities. This symbiotic relationship can lead to more efficient, accessible, and high-quality healthcare delivery.

## Conclusion and recommendations for implementation

ChatGPT demonstrates significant potential in healthcare while requiring careful implementation considerations. Based on current evidence and identified limitations, we propose several key recommendations for successful integration.

Technical implementation should prioritize validation protocols for AI-generated content, particularly in clinical documentation. Healthcare organizations must establish multi-step verification processes for AI-assisted clinical decisions, with special attention to areas where visual medical data processing remains limited. This technical framework should evolve alongside advances in AI capabilities.

For clinical integration, healthcare providers should develop clear guidelines defining appropriate use cases, focusing on tasks where ChatGPT has demonstrated proven efficacy. Regular monitoring of AI-assisted outcomes against traditional methods will ensure quality maintenance while providing data for continuous improvement. Organizations should maintain comprehensive documentation of AI tool usage in clinical settings to support both quality assurance and research efforts.

Professional development represents a crucial component of successful implementation. Healthcare institutions should invest in structured training programs that educate staff on both the capabilities and limitations of AI tools. This education should include practical experience under experienced mentors, ensuring competent and responsible AI tool usage across all levels of healthcare delivery.

Looking ahead, several development priorities emerge as critical for advancing ChatGPT’s healthcare applications. Enhanced visual data processing capabilities through multimodal AI integration will expand its utility in diagnostic fields. Improvements in emotional intelligence features will better support patient interactions, while specialized medical knowledge bases will increase accuracy across different healthcare specialties.

These recommendations provide a framework for responsible ChatGPT integration while acknowledging current limitations. Future research should focus on validating these approaches across diverse healthcare settings and patient populations, ensuring that AI enhancement of healthcare delivery benefits all stakeholders while maintaining the highest standards of patient care.
